# SGLT2 Inhibitor Dapagliflozin Attenuates Cardiomyocyte Injury and Inflammation Induced by PI3Kα-Selective Inhibitor Alpelisib and Fulvestrant Under Hyperglycemia

**DOI:** 10.3390/ijms27083597

**Published:** 2026-04-17

**Authors:** Vincenzo Quagliariello, Massimiliano Berretta, Matteo Barbato, Fabrizio Maurea, Maria Laura Canale, Andrea Paccone, Irma Bisceglia, Andrea Tedeschi, Marino Scherillo, Jacopo Santagata, Stefano Oliva, Christian Cadeddu Dessalvi, Pietro Forte, Cristiana D’Ambrosio, Tiziana Di Matola, Regina Parmentola, Domenico Gabrielli, Nicola Maurea

**Affiliations:** 1 Division of Cardiology, Istituto Nazionale Tumori-IRCCS-Fondazione G. Pascale, 80131 Napoli, Italy; a.paccone@istitutotumori.na.it (A.P.); jacopo.santagata@istitutotumori.na.it (J.S.); pietro.forte@istitutotumori.na.it (P.F.); regina.parmentola@istitutotumori.na.it (R.P.); n.maurea@istitutotumori.na.it (N.M.); 2 Department of Clinical and Experimental Medicine, University of Messina, 98125 Messina, Italy; berrettama@gmail.com; 3 Radiology Division, University of Aquila, 67100 Aquila, Italy; fabriziomaurea1998@gmail.com; 4 U.O.C. Cardiologia, Ospedale Versilia, 55041 Lido di Camaiore (LU), Italy; marialaura.canale@uslnordovest.toscana.it; 5 Servizi Cardiologici Integrati, Dipartimento Cardio-Toraco-Vascolare, Azienda Ospedaliera San Camillo Forlanini, 00152 Roma, Italy; irmabisceglia@gmail.com; 6 Cardiology, “Guglielmo da Saliceto” Hospital, 29121 Piacenza, Italy; andrea.tedeschimd@gmail.com; 7 Cardiologia Interventistica e Unità di Terapia Intensiva Cardiologica (UTIC), A.O. San Pio, Presidio Ospedaliero Gaetano Rummo, 82100 Benevento, Italy; marino.scherillo@libero.it; 8 Cardio-Oncology Unit, IRCCS Istituto Tumori, “Giovanni Paolo II”, 70124 Bari, Italy; s.oliva@oncologico.bari.it; 9 Department of Medical Sciences and Public Health, University of Cagliari, 09124 Cagliari, Italy; cadedduc@unica.it; 10 Cardiology Division, “F. Veneziale”, Molise Regional Health Company (ASREM), 86170 Isernia, Italy; cristianadambrosio1@virgilio.it; 11 U.O.C. Biochimica Clinica, AORN Ospedali dei Colli-Monaldi-Cotugno-CTO, 80131 Napoli, Italy; tizianadimatola7@gmail.com; 12 U.O.C. Cardiologia, Dipartimento Cardio-Toraco-Vascolare, Azienda Ospedaliera San Camillo Forlanini, Roma—Fondazione per il Tuo Cuore—Heart Care Foundation, 50121 Firenze, Italy; dgabrielli@scamilloforlanini.rm.it

**Keywords:** cardioncology, cancer, gliflozins, cardiotoxicity, heart failure, hyperglycemia

## Abstract

Activating PIK3CA mutations occur in approximately 40% of hormone receptor-positive (HR+)/HER2-negative breast cancers and represent a major driver of endocrine resistance. The PI3Kα-selective inhibitor alpelisib, in combination with fulvestrant, significantly improves progression-free survival in patients with PIK3CA-mutant disease, as demonstrated in the SOLAR-1 trial. However, this therapeutic strategy is frequently complicated by treatment-induced hyperglycemia, a metabolic disturbance that promotes oxidative stress, mitochondrial dysfunction, and inflammatory signaling, thereby increasing cardiovascular vulnerability. Sodium–glucose cotransporter-2 (SGLT2) inhibitors have emerged as cardiometabolic modulators with benefits extending beyond glucose lowering. In this study, we used a human cardiomyocyte in vitro model designed to recapitulate the hyperglycemic metabolic milieu observed in breast cancer patients receiving PI3Kα-targeted therapy, to investigate whether the SGLT2 inhibitor dapagliflozin directly protects cardiomyocytes from alpelisib- and fulvestrant-induced injury. Human induced pluripotent stem cell-derived cardiomyocytes (hiPSC-CMs) were cultured under hyperglycemic conditions (25 mM glucose) to mimic the metabolic environment associated with PI3Kα inhibitor-induced dysglycemia. Cells were exposed to alpelisib (100 nM) and fulvestrant (100 nM), alone or in combination, in the absence or presence of dapagliflozin (1 μM). Cardiomyocyte viability was assessed using the MTS assay, mitochondrial function by TMRM-based mitochondrial membrane potential (ΔΨm) measurements, and apoptosis by caspase-3 quantification. Cardiomyocyte injury was evaluated by release of cardiac troponin I and heart-type fatty acid binding protein (H-FABP). Lipid peroxidation markers (MDA and 4-HNE) were measured to assess oxidative membrane damage. Intracellular inflammasome-related signaling (NLRP3 and MyD88) and secreted inflammatory mediators (IL-1β, IL-18, IL-6, TNF-α, and CCL2) were quantified by ELISA. Exposure to alpelisib, particularly in combination with fulvestrant, significantly reduced cardiomyocyte viability, induced mitochondrial depolarization, and increased caspase-3-mediated apoptotic signaling. These alterations were accompanied by elevated lipid peroxidation (MDA and 4-HNE) and increased release of cardiac injury biomarkers (troponin I and H-FABP). Alpelisib-based treatments also activated inflammasome-related signaling, as indicated by increased intracellular NLRP3 and MyD88 levels and enhanced secretion of pro-inflammatory mediators (IL-1β, IL-18, IL-6, TNF-α, and CCL2). Co-treatment with dapagliflozin significantly attenuated these alterations, preserving mitochondrial membrane potential, reducing apoptotic signaling, limiting oxidative membrane damage, and suppressing inflammatory cytokine release. This study provides evidence that alpelisib-based therapy under hyperglycemic conditions is associated with oxidative, mitochondrial, and inflammatory stress responses in human cardiomyocytes, recapitulating key features of cardiometabolic stress relevant to PI3Kα-targeted therapy. Importantly, dapagliflozin markedly attenuated these alterations, supporting a potential cardioprotective role that may extend beyond glycemic control. These findings provide a mechanistic rationale for further investigation of SGLT2 inhibition as a cardiometabolic protective strategy in patients receiving PI3Kα inhibitor-based cancer therapy.

## 1. Introduction

The phosphatidylinositol-3-kinase (PI3K) signaling axis is a central regulator of cellular metabolism, growth, and survival, and its dysregulation represents a defining hallmark of multiple human malignancies [1,2]. In hormone receptor-positive (HR+)/HER2-negative breast cancer, activating mutations in PIK3CA occur in approximately 40% of cases and drive oncogenic addiction to PI3Kα-dependent signaling, contributing to endocrine resistance and disease progression [3]. Pharmacologic targeting of this pathway has therefore emerged as a rational therapeutic strategy, culminating in the clinical approval of the PI3Kα-selective inhibitor alpelisib in combination with fulvestrant [4]. While this regimen confers a significant improvement in progression-free survival, its clinical benefit is tempered by a distinctive and frequent metabolic toxicity profile, dominated by treatment-induced hyperglycemia [5].

In addition to metabolic effects, emerging preclinical evidence suggests that PI3Kα inhibition may exert direct electrophysiological effects on cardiomyocytes. Specifically, pharmacological inhibition of PI3Kα, including with alpelisib, has been shown to increase late sodium current (I_na_L), prolong action potential duration, and promote arrhythmogenic activity, supporting a potential direct mechanism of cardiotoxicity independent of systemic metabolic alterations. Hyperglycemia associated with PI3Kα inhibition is not a benign, on-target adverse event. Mechanistically, blockade of PI3Kα in insulin-responsive tissues disrupts physiological insulin signaling, resulting in acute insulin resistance, compensatory hyperinsulinemia, and sustained elevations in circulating glucose [6]. This metabolic derangement has been implicated not only in reduced treatment tolerability and premature drug discontinuation, but also in paradoxical oncogenic signaling reactivation through insulin-mediated engagement of residual PI3K/AKT pathways [7]. Beyond its oncologic implications, chronic or acute hyperglycemia is a well-established driver of cardiovascular injury, promoting oxidative stress, mitochondrial dysfunction, inflammatory signaling, and apoptotic cell death within cardiomyocytes. In the evolving field of cardio-oncology, such therapy-induced metabolic toxicity represents an emerging and underexplored mechanism of cancer treatment-related cardiovascular disease [8].

Notably, estrogen receptor antagonism with fulvestrant, while not classically cardiotoxic, may further exacerbate myocardial vulnerability by impairing estrogen-mediated cardioprotective signaling, particularly in metabolically stressed conditions [9]. Estrogen signaling has been shown to modulate mitochondrial biogenesis, antioxidant defenses, and glucose utilization in the heart; thus, combined PI3Kα inhibition and estrogen deprivation may synergistically compromise cardiomyocyte homeostasis [10]. However, despite the widespread clinical use of alpelisib and fulvestrant, their direct effects on human cardiomyocytes, especially under hyperglycemic conditions that recapitulate the clinical metabolic milieu, remain largely uncharacterized. In particular, whether the combination of PI3Kα inhibition, estrogen receptor blockade, and hyperglycemia may synergistically exacerbate cardiomyocyte vulnerability through both metabolic and direct cellular mechanisms remains an open and clinically relevant question. Whether cardiomyocyte injury arises solely from systemic metabolic perturbations or also from direct, cell-autonomous drug effects is a critical unanswered question with direct translational relevance [11].

**Figure 1:**
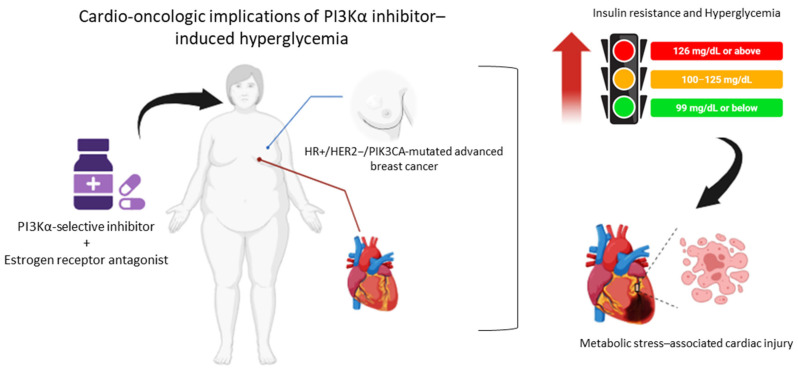
Cardio-metabolic vulnerability associated with PI3Kα inhibition in HR+/HER2− PIK3CA-mutant breast cancer. In patients receiving alpelisib combined with endocrine therapy, inhibition of insulin–PI3K signaling induces insulin resistance and hyperglycemia, which may amplify oxidative stress, inflammatory activation, and myocardial susceptibility to injury. This clinical scenario creates a cardio-oncologic vulnerability state that may predispose to cardiovascular adverse events and underscores the need for integrated cardiometabolic protection strategies.

Sodium–glucose cotransporter-2 (SGLT2) inhibitors have recently transformed the management of type 2 diabetes mellitus and heart failure, demonstrating robust cardioprotective effects that extend beyond glucose lowering [12]. Large cardiovascular outcome trials have consistently shown reductions in heart failure hospitalization and cardiovascular mortality, even in non-diabetic populations, implicating pleiotropic mechanisms such as improved myocardial energetics, attenuation of oxidative stress, modulation of intracellular sodium and calcium handling, and suppression of inflammatory and apoptotic pathways [13]. In the oncologic setting, emerging retrospective data suggest that SGLT2 inhibitors may represent an optimal strategy to counteract PI3K inhibitor-induced hyperglycemia, potentially prolonging exposure to targeted therapy and improving clinical outcomes [14,15]. Nevertheless, whether SGLT2 inhibition can directly protect cardiomyocytes from cancer therapy-induced injury, independent of systemic glucose control, remains unknown. Human induced pluripotent stem cell-derived cardiomyocytes (hiPSC-CMs) provide a powerful translational platform to dissect drug-induced cardiotoxicity under controlled metabolic conditions, allowing direct interrogation of mitochondrial function, oxidative stress, and apoptotic signaling in human cardiac cells [16,17]. Leveraging this model enables mechanistic insight into the intersection between targeted cancer therapy, hyperglycemia, and myocardial injury—an intersection that is increasingly relevant as cancer survivorship improves and long-term cardiovascular complications emerge as a dominant cause of morbidity [18]. This study aimed to evaluate the direct cardiotoxic effects of combined alpelisib and fulvestrant exposure on human cardiomyocytes under hyperglycemic conditions relevant to HR+/HER2− breast cancer therapy. In addition, it sought to determine whether dapagliflozin mitigates treatment-induced cardiac injury by preserving mitochondrial function and limiting oxidative, apoptotic, and inflammatory stress, thereby providing a mechanistic basis for SGLT2 inhibitor-mediated cardioprotection in the cardio-oncology setting (Figure 1).

## 2. Results

### 2.1. Dapagliflozin Prevents Cardiomyocyte Injury and Mitochondrial Damages Induced by Alpelisib and Fulvestrant Under Hyperglycemia and Normoglycemia

**Figure 2:**
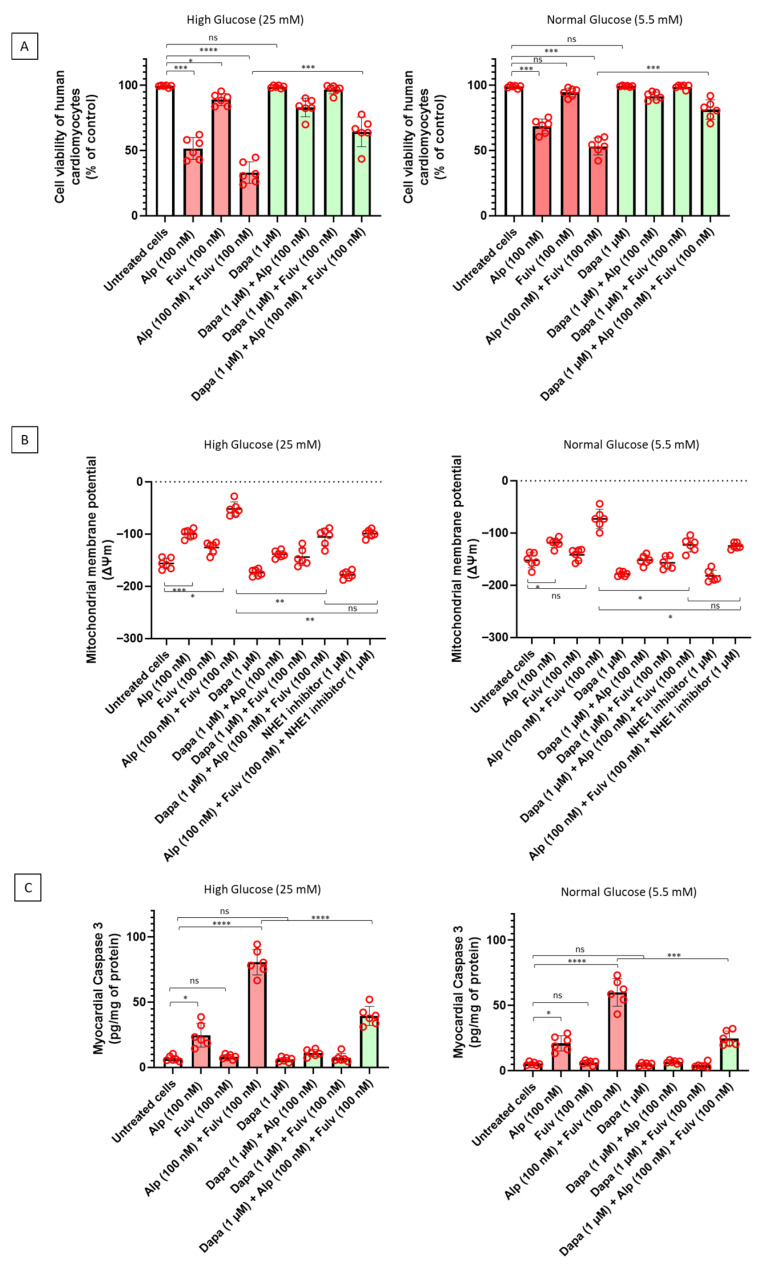
Effects of alpelisib, fulvestrant, and dapagliflozin on cardiomyocyte viability, mitochondrial membrane potential, and apoptotic signaling under hyperglycemic and normoglycemic conditions. Human iPSC-derived cardiomyocytes were treated for 24 h with alpelisib (100 nM), fulvestrant (100 nM), or their combination, in the absence or presence of dapagliflozin (1 µM), under hyperglycemic (25 mM glucose) or normoglycemic (5.5 mM glucose) conditions. (**A**) Cell viability was assessed by MTS assay and expressed as percentage relative to that of untreated control cells. (**B**) Mitochondrial membrane potential (ΔΨm) was measured using the TMRM method and expressed as relative changes in fluorescence. (**C**) Caspase-3 activity, a marker of execution-phase apoptosis, was quantified by luminescent assay and normalized to total protein content. Drug treatments were associated with reduced viability, mitochondrial depolarization, and increased apoptotic signaling, with more pronounced effects under hyperglycemic conditions. Dapagliflozin attenuated these alterations across conditions. Data are presented as mean ± SEM with individual data points representing independent biological replicates (n = 6). Statistical analysis was performed using two-way ANOVA followed by Tukey’s multiple-comparison test. * *p* < 0.05, ** *p* < 0.01, *** *p* < 0.001, **** *p* < 0.0001; ns, not significant.

To investigate whether PI3Kα inhibition and estrogen receptor blockade are associated with cardiomyocyte injury under different metabolic conditions, human iPSC-derived cardiomyocytes were exposed to alpelisib, fulvestrant, or their combination in the absence or presence of dapagliflozin under both hyperglycemic (25 mM) and normoglycemic (5.5 mM) conditions. As shown in Figure 2A, treatment with alpelisib was associated with a significant reduction in cardiomyocyte viability compared with untreated cells, while fulvestrant alone induced a more moderate effect. Combined exposure to alpelisib and fulvestrant resulted in a more pronounced reduction in viability, consistent with an additive impairment of cardiomyocyte metabolic activity. Notably, these effects were more evident under hyperglycemic conditions, whereas under normoglycemia the reduction in viability was attenuated. Co-treatment with dapagliflozin significantly improved cell viability across conditions, with a more marked effect observed in hyperglycemia. Given the central role of mitochondrial integrity in cardiomyocyte function, mitochondrial membrane potential (ΔΨm) was assessed using the TMRM assay (Figure 2B). Exposure to alpelisib was associated with mitochondrial depolarization, which was further accentuated in the presence of fulvestrant. This effect was more pronounced under hyperglycemic conditions, indicating increased mitochondrial susceptibility in a high-glucose environment. Dapagliflozin co-treatment attenuated mitochondrial depolarization, partially restoring ΔΨm toward baseline levels. To evaluate activation of apoptotic signaling, caspase-3 activity was measured as a marker of execution-phase apoptosis (Figure 2C). Alpelisib increased caspase-3 activation compared with untreated cells, with the highest levels observed in the combined alpelisib and fulvestrant condition. Consistent with the effects observed on viability and mitochondrial function, apoptotic activation was more pronounced under hyperglycemic conditions. Dapagliflozin significantly reduced caspase-3 activity in drug-treated cardiomyocytes, indicating attenuation of apoptosis-associated signaling. Collectively, these findings indicate that PI3Kα inhibition and estrogen receptor blockade are associated with cardiomyocyte injury characterized by reduced viability, mitochondrial dysfunction, and activation of apoptotic pathways, with hyperglycemia acting as a key amplifying factor. Dapagliflozin attenuates these alterations, supporting a cardioprotective effect at the level of mitochondrial and apoptotic signaling. Notably, hyperglycemia alone induced a modest reduction in cardiomyocyte viability, supporting the presence of a basal metabolic stress condition that amplifies drug-induced injury, while the ability of dapagliflozin to confer protection under both normoglycemic and hyperglycemic conditions further suggests a direct cardiomyocyte-targeted effect beyond glucose lowering.

### 2.2. Dapagliflozin Reduces Cardiac Troponin and H-FABP Release

**Figure 3:**
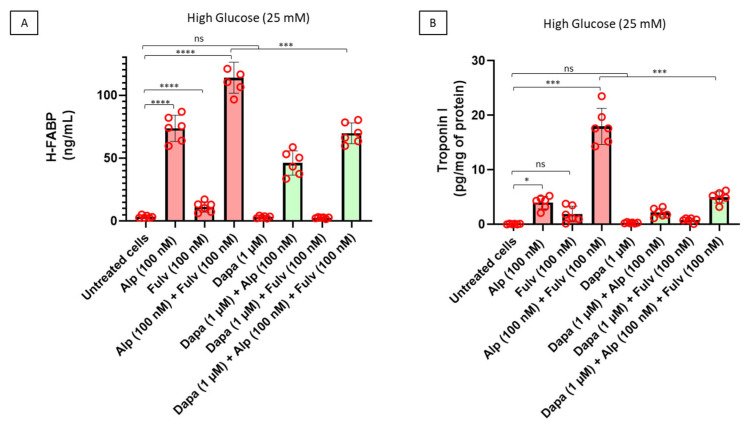
Alpelisib and fulvestrant induce cardiomyocyte injury, attenuated by dapagliflozin co-treatment. Human iPSC-derived cardiomyocytes were treated for 24 h with alpelisib (100 nM), fulvestrant (100 nM), or their combination, in the absence or presence of dapagliflozin (1 µM) under hyperglycemic conditions. (**A**) Heart-type fatty acid binding protein (H-FABP) and (**B**) cardiac troponin I levels were quantified in culture supernatants as markers of cardiomyocyte injury and normalized to total protein content where applicable. Drug treatments were associated with increased biomarker release, while dapagliflozin reduced these responses. Data are presented as mean ± SEM with individual data points representing independent biological replicates (n = 6). Statistical analysis was performed using two-way ANOVA followed by Tukey’s multiple-comparison test. * *p* < 0.05, *** *p* < 0.001, **** *p* < 0.0001; ns, not significant.

To further characterize the extent of cardiomyocyte injury induced by oncologic treatment under hyperglycemic conditions, we quantified the extracellular release of myocardial injury biomarkers, including heart-type fatty acid binding protein (H-FABP) and cardiac troponin I. As shown in Figure 3A, treatment with alpelisib significantly increased H-FABP release into the culture medium compared with untreated cardiomyocytes, indicating early membrane injury and cytosolic protein leakage. Fulvestrant alone induced only a modest increase in H-FABP levels, whereas the combination of alpelisib and fulvestrant resulted in a marked amplification of H-FABP release, suggesting enhanced cardiomyocyte injury under combined PI3Kα inhibition and estrogen receptor blockade. Importantly, co-treatment with dapagliflozin significantly reduced H-FABP release in cardiomyocytes exposed to oncologic drugs, indicating attenuation of drug-induced cellular damage. Consistent with these findings, measurement of cardiac troponin I, a highly specific marker of cardiomyocyte structural injury, revealed a similar pattern (Figure 3B). Exposure to alpelisib significantly increased troponin I release compared with untreated cells, while fulvestrant alone produced a smaller increase. Notably, combined treatment with alpelisib and fulvestrant induced the highest levels of troponin I release, reflecting a greater degree of cardiomyocyte injury. In contrast, dapagliflozin co-treatment significantly reduced troponin I release across drug-treated conditions, indicating a protective effect against oncologic therapy-associated cardiomyocyte damage. Collectively, these findings demonstrate that PI3Kα inhibition and estrogen receptor blockade promote cardiomyocyte injury characterized by increased release of canonical myocardial damage biomarkers. Importantly, dapagliflozin markedly limits this injury phenotype, supporting its potential role in mitigating cardiotoxic effects associated with targeted cancer therapies under hyperglycemic conditions.

### 2.3. Dapagliflozin Mitigates Oxidative Membrane Injury and Electrophysiological Alterations Induced by Alpelisib and Fulvestrant

**Figure 4:**
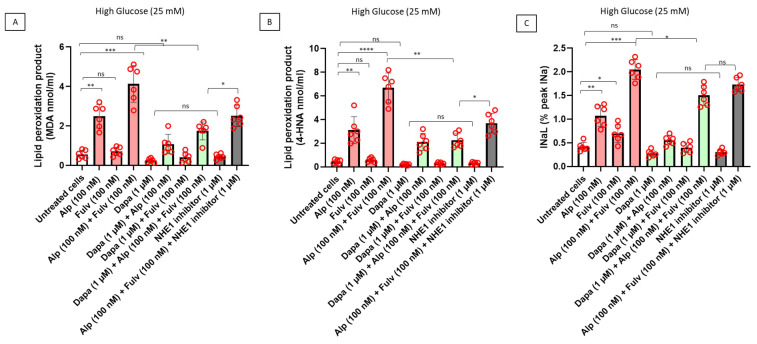
Dapagliflozin and NHE1 inhibition differentially modulate oxidative stress and electrophysiological alterations in human cardiomyocytes exposed to PI3Kα inhibition under hyperglycemic conditions. Human iPSC-derived cardiomyocytes were treated for 24 h with alpelisib (100 nM), fulvestrant (100 nM), or their combination, in the absence or presence of dapagliflozin (1 µM) or a selective NHE1 inhibitor under hyperglycemic conditions. (**A**) Lipid peroxidation was assessed by measuring malondialdehyde (MDA) levels in cardiomyocyte lysates. (**B**) 4-hydroxynonenal (4-HNE) levels were quantified as an additional marker of oxidative lipid damage. (**C**) Late sodium current (I_na_L), expressed as a percentage of peak sodium current, was measured to evaluate electrophysiological alterations. Drug treatments were associated with increased oxidative stress and I_na_L. Both dapagliflozin and NHE1 inhibition reduced these alterations, with dapagliflozin showing a more pronounced effect. Data are presented as mean ± SEM with individual data points representing independent biological replicates (n = 6). Statistical analysis was performed using two-way ANOVA followed by Tukey’s multiple-comparison test. * *p* < 0.05, ** *p* < 0.01, *** *p* < 0.001, **** *p* < 0.0001; ns, not significant.

Given the central role of oxidative stress in cardiotoxicity associated with metabolic and oncologic therapies, we next evaluated whether alpelisib and fulvestrant are associated with oxidative membrane damage in cardiomyocytes under hyperglycemic conditions and whether these effects are modulated by dapagliflozin and NHE1 inhibition. As shown in Figure 4A, treatment with alpelisib was associated with increased intracellular levels of malondialdehyde (MDA), a marker of lipid peroxidation, compared with untreated cardiomyocytes. Fulvestrant alone induced a more modest effect, whereas combined exposure to alpelisib and fulvestrant resulted in a greater increase in MDA levels. Co-treatment with dapagliflozin markedly reduced MDA accumulation, while pharmacological inhibition of NHE1 also attenuated lipid peroxidation, although to a lesser extent. Consistent with these findings, quantification of 4-hydroxynonenal (4-HNE), another product of oxidative lipid damage, revealed a similar pattern (Figure 4B). Alpelisib increased 4-HNE levels relative to those of untreated cells, with the highest levels observed following combined treatment with fulvestrant. Both dapagliflozin and NHE1 inhibition reduced 4-HNE accumulation, with a more pronounced effect observed in the presence of dapagliflozin. Given the link between oxidative stress and electrophysiological alterations, late sodium current (I_na_L) was assessed (Figure 4C). Treatment with alpelisib was associated with increased I_na_L, which was further enhanced in the combined alpelisib and fulvestrant condition. Both dapagliflozin and NHE1 inhibition attenuated this increase, supporting a role for intracellular Na^+^ handling in cardiomyocyte electrical remodeling. Notably, the reduction in I_na_L was more pronounced with dapagliflozin. Collectively, these findings indicate that PI3Kα inhibition and estrogen receptor blockade are associated with increased oxidative membrane damage and electrophysiological alterations in cardiomyocytes under hyperglycemic conditions. Both dapagliflozin and NHE1 inhibition mitigate these effects, although the greater efficacy of dapagliflozin suggests that additional mechanisms beyond NHE1 modulation may contribute to its cardioprotective profile.

### 2.4. Dapagliflozin Suppresses Inflammasome Activation and Pro-Inflammatory Cytokine Release

**Figure 5:**
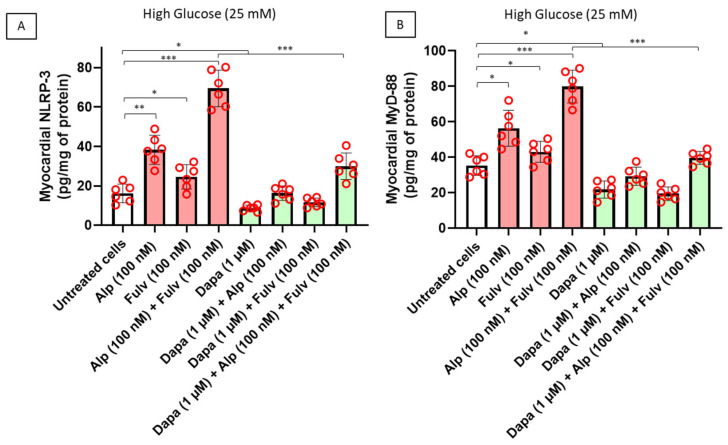
PI3Kα inhibition and estrogen receptor blockade activate inflammasome-related signaling in cardiomyocytes, attenuated by dapagliflozin. Human iPSC-derived cardiomyocytes were treated for 24 h with alpelisib (100 nM), fulvestrant (100 nM), or their combination, in the absence or presence of dapagliflozin (1 µM) under hyperglycemic conditions. Intracellular levels of (**A**) NLRP3 and (**B**) MyD88 were quantified in cardiomyocyte lysates and normalized to total protein content. Drug treatments were associated with increased expression of inflammasome-related signaling components, while dapagliflozin reduced these responses. Data are presented as mean ± SEM with individual data points representing independent biological replicates (n = 6). Statistical analysis was performed using two-way ANOVA followed by Tukey’s multiple-comparison test. * *p* < 0.05, ** *p* < 0.01, *** *p* < 0.001.

Because oxidative stress and mitochondrial dysfunction are well-recognized triggers of innate inflammatory signaling in cardiomyocytes, we next investigated whether treatment with alpelisib and fulvestrant activates inflammasome-related pathways under hyperglycemic conditions. As shown in Figure 5A, treatment with alpelisib significantly increased intracellular levels of NLRP3, a central component of the inflammasome complex, compared with untreated cardiomyocytes. Fulvestrant alone induced a moderate increase in NLRP3 expression, whereas the combination of alpelisib and fulvestrant resulted in a markedly stronger activation of inflammasome signaling. These findings indicate that concurrent PI3Kα inhibition and estrogen receptor blockade amplify inflammatory stress responses in cardiomyocytes exposed to hyperglycemic conditions. Importantly, co-treatment with dapagliflozin significantly reduced NLRP3 expression in drug-treated cardiomyocytes, suggesting suppression of inflammasome activation. To further characterize inflammatory signaling pathways, we quantified MyD88, a key adaptor protein involved in Toll-like receptor-dependent inflammatory responses. As illustrated in Figure 5B, exposure to alpelisib significantly increased MyD88 levels relative to those of untreated cells, while fulvestrant alone produced a smaller effect. Combined treatment with alpelisib and fulvestrant induced the highest MyD88 expression, consistent with enhanced activation of innate inflammatory signaling pathways. Notably, dapagliflozin co-treatment significantly reduced MyD88 expression across drug-treated groups, indicating inhibition of cardiomyocyte inflammatory signaling. Taken together, these results demonstrate that PI3Kα inhibition and estrogen receptor blockade promote activation of inflammasome-associated pathways in cardiomyocytes under hyperglycemic stress. Dapagliflozin markedly suppresses these inflammatory responses, supporting a cardioprotective mechanism involving modulation of innate immune signaling in cardiac cells.

**Figure 6:**
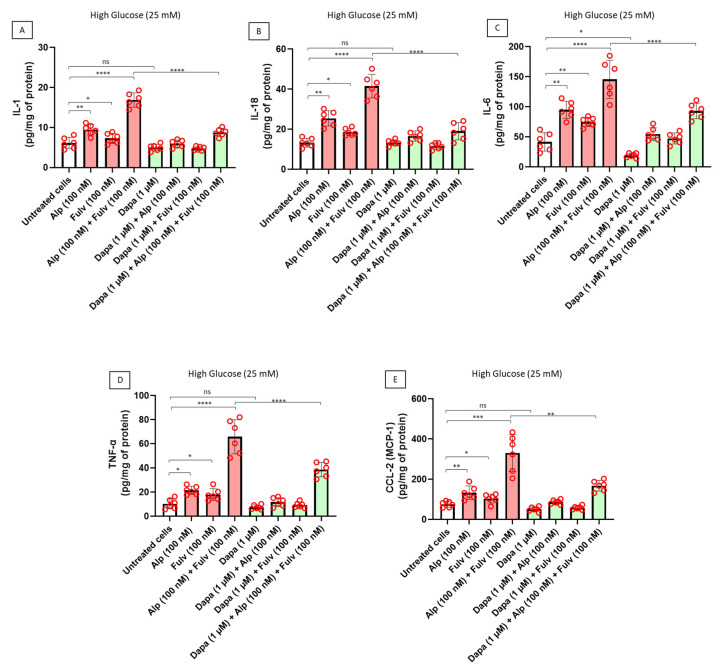
Dapagliflozin suppresses PI3Kα inhibitor-associated inflammatory cytokine signaling in human cardiomyocytes under hyperglycemic stress. Human iPSC-derived cardiomyocytes were treated for 24 h with alpelisib (100 nM), fulvestrant (100 nM), or their combination, in the absence or presence of dapagliflozin (1 µM) under hyperglycemic conditions. Intracellular levels of (**A**) IL-1β and (**B**) IL-18, and secreted levels of (**C**) IL-6, (**D**) TNF-α, and (**E**) CCL2 were quantified and normalized to total protein content. Alpelisib and combined treatment were associated with increased inflammatory signaling, while dapagliflozin reduced cytokine levels across conditions. Data are presented as mean ± SEM with individual data points representing independent biological replicates (n = 6). Statistical analysis was performed using two-way ANOVA followed by Tukey’s multiple-comparison test. * *p* < 0.05, ** *p* < 0.01, *** *p* < 0.001, **** *p* < 0.0001; ns, not significant.

Moreover, to determine whether activation of inflammasome-related pathways translated into downstream inflammatory signaling, we next quantified the expression of key pro-inflammatory cytokines and chemokines in cardiomyocytes exposed to alpelisib and fulvestrant under hyperglycemic conditions. As shown in Figure 6A, treatment with alpelisib significantly increased intracellular levels of IL-1β, a canonical inflammasome-dependent cytokine, compared with untreated cardiomyocytes. Fulvestrant alone induced only a modest increase in IL-1β expression, whereas combined exposure to alpelisib and fulvestrant resulted in a more pronounced inflammatory response. Co-treatment with dapagliflozin significantly reduced IL-1β levels in drug-treated cardiomyocytes. A similar pattern was observed for IL-18, another cytokine closely associated with inflammasome activation (Figure 6B). Alpelisib significantly increased IL-18 levels relative to those of untreated cells, while the combination of alpelisib and fulvestrant further amplified this response. Dapagliflozin co-treatment markedly reduced IL-18 expression across drug-treated conditions. We next evaluated IL-6, a key mediator of inflammatory and stress-related signaling in cardiomyocytes. As illustrated in Figure 6C, alpelisib significantly increased IL-6 levels, with a substantially greater increase observed in the combined alpelisib and fulvestrant condition. Importantly, dapagliflozin significantly attenuated IL-6 production in cardiomyocytes exposed to oncologic therapies. Consistent with these findings, TNF-α, a central cytokine involved in cardiomyocyte inflammatory injury, was significantly increased following treatment with alpelisib and fulvestrant (Figure 6D). The highest TNF-α levels were observed in the combination group, whereas dapagliflozin significantly reduced TNF-α expression in drug-treated cardiomyocytes. Finally, we quantified CCL2 (MCP-1), a chemokine involved in inflammatory cell recruitment and myocardial stress signaling. As shown in Figure 6E, alpelisib significantly increased CCL2 expression compared with untreated cells, while combined exposure to alpelisib and fulvestrant produced the strongest increase. Dapagliflozin co-treatment significantly reduced CCL2 levels, indicating suppression of chemokine-mediated inflammatory signaling. Collectively, these findings demonstrate that PI3Kα inhibition and estrogen receptor blockade induce a broad pro-inflammatory cytokine program in cardiomyocytes under hyperglycemic stress. Dapagliflozin markedly suppresses this inflammatory signaling cascade, supporting a cardioprotective mechanism involving modulation of inflammasome-dependent cytokine responses.

## 3. Discussion

**Figure 7:**
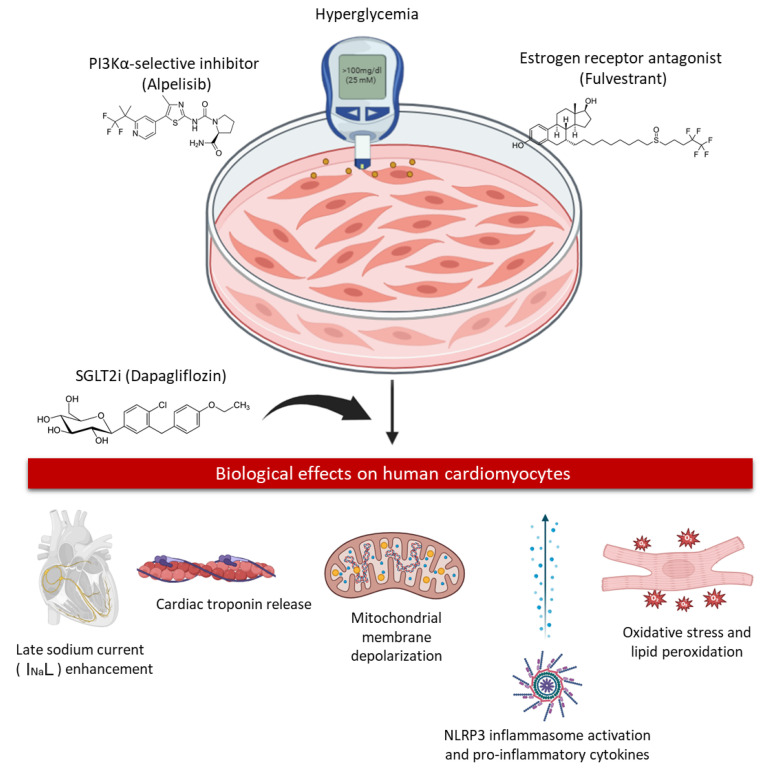
Experimental design and conceptual overview of hyperglycemia-associated cardiomyocyte stress induced by PI3Kα inhibition and estrogen receptor blockade, and its modulation by dapagliflozin. Human iPSC-derived cardiomyocytes (hiPSC-CMs) were cultured under hyperglycemic conditions (25 mM glucose) and exposed for 24 h to the PI3Kα-selective inhibitor alpelisib (100 nM) and/or the estrogen receptor antagonist fulvestrant (100 nM), in the absence or presence of the SGLT2 inhibitor dapagliflozin (1 µM). Combined PI3Kα inhibition and estrogen receptor blockade under hyperglycemia were associated with a cardiomyocyte injury phenotype characterized by increased late sodium current (I_Na_L), cardiac troponin release, mitochondrial membrane depolarization (ΔΨm loss), oxidative stress and lipid peroxidation, and inflammasome-associated inflammatory signaling. Dapagliflozin attenuated these alterations and was associated with preservation of mitochondrial integrity and redox homeostasis. The schematic provides a conceptual representation of interconnected pathways rather than a strictly linear mechanistic cascade and summarizes the experimental model and the principal biological endpoints assessed in human cardiomyocytes.

In the present study, we provide evidence that the combined inhibition of PI3Kα signaling and estrogen receptor blockade is associated with a cardiotoxic phenotype in human cardiomyocytes exposed to hyperglycemic conditions. Specifically, treatment with the PI3Kα-selective inhibitor alpelisib and the estrogen receptor antagonist fulvestrant was associated with mitochondrial dysfunction, oxidative membrane injury, electrophysiological alterations, inflammasome-associated signaling, and pro-inflammatory cytokine signaling [19,20]. Importantly, co-treatment with the SGLT2 inhibitor dapagliflozin markedly attenuated these pathological responses, preserving cardiomyocyte viability and limiting both oxidative and inflammatory stress pathways. These findings suggest a previously underappreciated cell-autonomous component of cardiotoxicity associated with PI3Kα-targeted therapy, in which hyperglycemia acts as a central amplifier of myocardial vulnerability. The conceptual framework emerging from our experimental model, summarized in Figure 7, indicates that combined PI3Kα inhibition and estrogen receptor blockade under hyperglycemic conditions are associated with a network of alterations including mitochondrial depolarization, lipid peroxidation, enhancement of late sodium current (I_na_L), inflammasome-related signaling, and release of cardiac injury biomarkers [21,22]. Importantly, these processes are likely to operate through interconnected and mutually reinforcing pathways rather than a strictly linear cascade. Dapagliflozin appears to modulate multiple nodes within this network, restoring redox balance and suppressing inflammatory signaling in cardiomyocytes. Hyperglycemia is increasingly recognized not merely as a metabolic adverse effect of targeted cancer therapies but as a biologically active driver of tissue injury [23,24]. In cardiomyocytes, elevated glucose availability promotes mitochondrial substrate overload, leading to electron transport chain dysfunction, increased electron leakage, and excessive production of reactive oxygen species (ROS) [25]. This redox imbalance triggers multiple downstream stress pathways, including lipid peroxidation, mitochondrial membrane depolarization, and activation of apoptosis-related signaling [26]. Consistent with this paradigm, our data demonstrate a marked increase in lipid peroxidation markers (MDA and 4-HNE), mitochondrial membrane potential loss, and caspase-3 activation in cardiomyocytes exposed to alpelisib and fulvestrant under hyperglycemic conditions.

Mechanistically, PI3Kα inhibition is known to disrupt insulin-mediated glucose uptake and Akt-dependent survival signaling, thereby impairing metabolic flexibility and cellular stress responses in cardiomyocytes [27]. Although PI3K/AKT signaling and insulin-dependent pathways represent central mechanisms linking PI3Kα inhibition to cardiometabolic dysregulation, these pathways were not directly interrogated in the present study and should therefore be considered as plausible, literature-supported contributors rather than experimentally demonstrated mechanisms. Future investigations using targeted molecular approaches will be required to define the precise role of PI3K/AKT signaling and insulin regulation in mediating the cardioprotective effects of dapagliflozin. In parallel, estrogen receptor blockade may reduce estrogen-dependent cardioprotective signaling pathways, including mitochondrial biogenesis, antioxidant defense regulation, and calcium handling stability [28,29]. Estrogen signaling is known to enhance mitochondrial respiratory efficiency and activate transcriptional programs associated with antioxidant protection [30]. Consequently, the simultaneous pharmacologic inhibition of PI3Kα and estrogen receptor signaling may contribute to a state of metabolic vulnerability, particularly under hyperglycemic conditions.

In addition to oxidative damage, our findings highlight a potential role for innate immune signaling in PI3Kα inhibitor-associated cardiotoxicity. Hyperglycemia-induced mitochondrial ROS production is a potent trigger of the NLRP3 inflammasome, a cytosolic multiprotein complex that integrates metabolic stress signals and orchestrates sterile inflammatory responses [31,32,33]. In the present study, our findings reflect activation of inflammasome-associated molecular components rather than definitive demonstration of canonical inflammasome activation. Cardiomyocytes exposed to alpelisib and fulvestrant displayed increased expression of NLRP3 and MyD88, accompanied by elevated levels of downstream inflammatory mediators including IL-1β, IL-18, IL-6, TNF-α, and CCL2 [34]. These observations are consistent with engagement of inflammasome-related inflammatory signaling pathways, although further studies are required to confirm functional inflammasome assembly in this context. Notably, although canonical inflammasome activation is more extensively characterized in immune cells, increasing evidence indicates that cardiomyocytes can express NLRP3 and related inflammasome components under pathological stress conditions, including exposure to cardiotoxic agents such as doxorubicin. In this context, NLRP3 upregulation in cardiomyocytes has been associated with oxidative stress-driven inflammatory signaling, supporting the concept that cardiomyocytes may contribute to inflammasome-associated responses in non-immune settings. Importantly, this inflammatory phenotype was paralleled by electrophysiological alterations characterized by increased late sodium current (I_na_L), a well-established contributor to arrhythmogenic risk in cardiomyocytes [35,36]. Enhanced I_na_L promotes intracellular sodium accumulation, secondary calcium overload, and further mitochondrial dysfunction, thereby reinforcing the pathological interplay between oxidative stress, inflammation, and cardiomyocyte injury [37,38]. Consistent with this observation, previous experimental studies have demonstrated that pharmacological inhibition of PI3Kα, including with alpelisib, increases late sodium current and promotes arrhythmogenic activity in cardiomyocytes, supporting a potential direct electrophysiological mechanism of cardiotoxicity independent of systemic metabolic alterations [39]. A central and clinically relevant finding of this study is that dapagliflozin significantly attenuates these pathological processes at the cardiomyocyte level. Co-treatment with dapagliflozin restored mitochondrial membrane potential, reduced lipid peroxidation, modulated inflammasome-associated signaling, and significantly decreased the expression of inflammatory cytokines. These effects were observed in isolated cardiomyocytes, suggesting that the cardioprotective actions of dapagliflozin may involve direct cellular effects in addition to its known systemic metabolic actions [40].

Several mechanisms may contribute to this cardioprotective effect. SGLT2 inhibitors have been shown to improve myocardial energetics by shifting substrate utilization toward ketone bodies and fatty acids, thereby increasing mitochondrial efficiency and reducing oxidative burden [41,42,43,44,45]. In addition, dapagliflozin has been reported to reduce intracellular sodium accumulation through indirect inhibition of the cardiac Na^+^/H^+^ exchanger, which may contribute to the normalization of late sodium current observed in our study [46]. Improved intracellular sodium and calcium handling may in turn support mitochondrial function and reduce ROS generation, thereby limiting activation of inflammatory pathways [47].

From a translational perspective, these findings have important implications for the management of cardiometabolic toxicity in patients receiving PI3Kα inhibitors. Alpelisib-induced hyperglycemia is among the most common causes of treatment interruption in patients with HR+/HER2-negative breast cancer [48]. Conventional antidiabetic therapies primarily target systemic glycemic control but may not address the downstream cardiometabolic consequences of PI3Kα inhibition [49,50]. Our results suggest that SGLT2 inhibitors may represent a promising therapeutic strategy, as they improve metabolic homeostasis and may attenuate cardiomyocyte injury associated with oxidative and inflammatory stress. Furthermore, hyperinsulinemia secondary to PI3Kα inhibition has been shown to reactivate oncogenic PI3K/AKT signaling in tumor cells, potentially undermining the antitumor efficacy of targeted therapy [51,52,53]. By reducing insulin levels and improving metabolic regulation, SGLT2 inhibitors may therefore provide complementary benefits in precision oncology, including potential cardiometabolic protection alongside maintenance of oncologic pathway inhibition. Collectively, these observations support the concept that hyperglycemia establishes a pro-oxidative and pro-inflammatory milieu that amplifies cardiomyocyte susceptibility to pharmacological stress, potentially through activation of advanced glycation end product (AGE)–RAGE signaling and downstream pathways including NF-κB-mediated transcription, mitochondrial ROS generation, and inflammasome-associated responses. Therefore, hyperglycemia may act as a critical metabolic “second hit,” enhancing oxidative damage, inflammatory signaling, and electrophysiological instability, thereby exacerbating the cardiotoxic effects associated with PI3Kα inhibition and estrogen receptor blockade. Notably, several limitations of the present study should be acknowledged. First, although human iPSC-derived cardiomyocytes represent a highly relevant translational model, they do not fully recapitulate the structural and cellular complexity of the adult human myocardium [54]. Second, the present experiments were conducted under controlled in vitro conditions and therefore cannot account for systemic factors that influence cardiotoxicity in patients [55]. Third, while our data demonstrate modulation of oxidative stress and inflammatory signaling pathways, they do not establish direct causal molecular mechanisms, and further studies are required to define upstream regulatory pathways [56]. Finally, the electrophysiological alterations observed warrant further investigation using advanced in vitro and in vivo models to determine their functional relevance for arrhythmogenic risk [57,58]. An important limitation of the present study is the lack of in vivo validation, which limits the ability to fully capture the systemic nature of cardiometabolic toxicity associated with PI3Kα inhibition. While human iPSC-derived cardiomyocytes provide a relevant translational model to investigate cell-autonomous mechanisms, they do not account for circulating factors such as glucose, insulin, and neurohormonal regulation that may influence cardiotoxic responses. To address this, ongoing studies in our group are evaluating the effects of PI3Kα inhibition and SGLT2 blockade in animal models, with integrated cardiometabolic profiling, including circulating glucose and insulin levels and analysis of PI3K/AKT signaling pathways. In addition, cardiac structure and function will be assessed by high-resolution echocardiography (VEVO2100, Fujifilm; Tokyo, Japan), together with electrophysiological analyses. These studies will be essential to determine whether the cardioprotective effects of dapagliflozin observed in vitro translate into a physiologically relevant in vivo setting. Therefore, future studies integrating animal studies and prospective clinical investigations will be essential to determine whether the cardioprotective mechanisms identified in this work translate into improved cardiovascular outcomes in patients receiving PI3Kα-targeted therapies.

## 4. Materials and Methods

### 4.1. Cell Culture of Human iPSC-Derived Cardiomyocytes

Human induced pluripotent stem cell-derived cardiomyocytes (hiPSC-CMs; Ncardia, Cat. No. CMC-100, Cologne, Germany) were used as an in vitro model of human cardiomyocytes. Cryopreserved cells were thawed according to the manufacturer’s instructions and plated onto growth factor-reduced Matrigel-coated plates to ensure optimal cell attachment and maturation [59,60]. For viability and fluorescence-based assays, cells were seeded in 96-well plates at a density of 20,000 cells per well and maintained in Ncardia Maintenance Medium under standard culture conditions (37 °C, 5% CO_2_, ≥95% humidity). Culture medium was refreshed every 48 h. Following plating, cardiomyocytes were allowed to stabilize and recover for 7 days prior to pharmacological treatments, during which time cells developed synchronous spontaneous contractile activity and stable morphology. To reproduce the hyperglycemic metabolic environment associated with PI3Kα inhibitor-induced dysglycemia, cells were cultured under hyperglycemic conditions (25 mM D-glucose). This glucose concentration is widely used in cardiomyocyte models to mimic hyperglycemia-driven metabolic stress and has been associated with increased oxidative stress and mitochondrial dysfunction in cardiomyocytes [61]. After the stabilization period, hiPSC-CMs were exposed to the indicated pharmacological treatments under hyperglycemic conditions for the durations specified in each experiment.

### 4.2. Drugs, Preparation, and Concentration Selection

Alpelisib (Cat. No. AMBH303C46DC; Sigma Aldrich, Milan, Italy), Fulvestrant (Cat. No. I4409; Sigma Aldrich, Milan, Italy), and Dapagliflozin (Cat. No. SML2804; Sigma Aldrich, Milan, Italy) were purchased from certified commercial suppliers and prepared as 10 mM stock solutions in dimethyl sulfoxide (DMSO, Sigma Aldrich, Milan, Italy), aliquoted, and stored at −20 °C protected from light. Working solutions were freshly prepared in pre-warmed culture medium immediately prior to each experiment. The final DMSO concentration was kept constant across all experimental conditions (≤0.1% *v*/*v*), in accordance with established in vitro pharmacological standards [62]. Drug concentrations were selected based on clinically relevant exposure data and previously published in vitro mechanistic studies [63]. Alpelisib was used at 100 nM, a submicromolar concentration within the range supported by clinical pharmacokinetic data, where the reported unbound peak plasma exposure at the approved 300 mg/day dose is approximately 0.7 µM [64]. Fulvestrant was used at 100 nM, consistent with literature-based nanomolar ranges reflecting steady-state clinical C_max values (~15–16 ng/mL), which correspond to low-tens of nanomolar concentrations [65]. Dapagliflozin was administered at 1 µM, a concentration within the submicromolar–low micromolar range commonly employed in mechanistic cardiomyocyte studies and aligned with clinically observed exposure levels (reported C_max ~0.2 µM at the 10 mg dose) [66]. Together, these concentrations were selected to remain biologically meaningful, clinically anchored, and consistent with prior experimental literature investigating cardiometabolic and redox-related mechanisms.

### 4.3. Experimental Design and Treatment Groups

Following metabolic adaptation (hyperglycemia), iPSC-CMs were exposed to vehicle, alpelisib, fulvestrant, or the combination of alpelisib plus fulvestrant, in the absence or presence of dapagliflozin. Dapagliflozin was administered as a co-treatment (added 30–60 min before alpelisib/fulvestrant or concomitantly at time 0, and maintained throughout exposure). Treatments were conducted for 24 h. Notably, NHE1 inhibition was included as a mechanistic control to evaluate the contribution of intracellular Na^+^ handling to drug-induced cardiomyocyte dysfunction. Specifically, a selective Na^+^/H^+^ exchanger-1 (NHE1) inhibitor (cariporide 1 µM; Sigma Aldrich, Milan, Italy) was used to determine whether modulation of Na^+^ influx could reproduce the effects of dapagliflozin on mitochondrial membrane potential, oxidative stress (lipid peroxidation markers), and late sodium current (I_na_L). This approach allowed us to dissect the relative contribution of Na^+^-dependent mechanisms to the observed cardioprotective effects, and to distinguish NHE1-related effects from broader pleiotropic actions of SGLT2 inhibition. At each endpoint, supernatants were collected for biomarker and cytokine ELISAs, and cells were processed for viability, oxidative stress/peroxidation, mitochondrial function, apoptosis assays, and (where planned) protein extraction.

### 4.4. Cell Viability (MTS Assay)

Cell viability was assessed using an MTS-based colorimetric assay (CellTiter 96^®^ AQueous One Solution Cell Proliferation Assay, Promega, Mannheim, Germany; Cat. No. G3582), which provides an indirect measure of metabolically active viable cells. At the end of the treatment period, culture plates were equilibrated to room temperature for approximately 10 min. The MTS reagent was then added directly to each well at 10–20% of the culture volume, and cells were incubated at 37 °C for 1–3 h protected from light, according to the manufacturer’s instructions [67]. Absorbance was measured at 490 nm using a microplate reader. Background absorbance from cell-free wells containing culture medium and MTS reagent was subtracted from all measurements. Viability values were normalized to the corresponding vehicle-treated control within the same glucose condition in order to minimize potential baseline differences related to glucose exposure. Results are expressed as percentage of control viability.

### 4.5. Lipid Peroxidation: MDA and 4-HNE Content

Lipid peroxidation was evaluated in human iPSC-derived cardiomyocytes as an indicator of oxidative membrane damage by quantifying two established aldehydic end-products, malondialdehyde (MDA) and 4-hydroxy-2-nonenal (4-HNE) [68]. Following the experimental treatments, cardiomyocytes were harvested and lysed in ice-cold lysis buffer. After treatments, cell lysates were clarified by centrifugation to remove insoluble material, and supernatants were used for subsequent analyses. MDA levels were determined using a thiobarbituric acid reactive substances (TBARS)-based colorimetric assay (MDA Assay Kit, MAK085; Sigma-Aldrich, Milan, Italy) according to the manufacturer’s instructions. Briefly, cell lysates were combined with the thiobarbituric acid (TBA; Sigma Aldrich, Milan, Italy) reaction mixture and incubated at 95 °C for 60 min to allow formation of the MDA–TBA adduct. After cooling, the resulting chromogenic product was quantified spectrophotometrically at 532 nm using a microplate reader. To further assess lipid peroxidation, 4-HNE–protein adducts were measured using a human-specific ELISA-based lipid peroxidation assay (Lipid Peroxidation 4-HNE Assay Kit, ab238538; Abcam, Milan, Italy). Standards and samples were added to pre-coated 96-well plates and incubated according to the manufacturer’s protocol. After sequential incubation with anti-4-HNE detection antibody and substrate solution, absorbance was measured at 450 nm, and concentrations were calculated from a standard calibration curve [69]. Because oxidative stress measurements in cell culture can be influenced by variations in cell number and protein yield, MDA and 4-HNE values were normalized to total protein content determined from parallel lysates using a bicinchoninic acid (BCA) protein assay (Sigma Aldrich, Milan, Italy). Data are therefore expressed relative to total cellular protein content to account for differences in sample loading and cell density across experimental conditions. All measurements were performed in duplicate using independent biological replicates.

### 4.6. Inflammasome Signaling and Inflammatory Cytokine Profiling

To investigate inflammatory signaling pathways associated with cardiomyocyte injury, intracellular inflammasome-related proteins and secreted inflammatory mediators were quantified in human induced pluripotent stem cell-derived cardiomyocytes (hiPSC-CMs) exposed to experimental treatments. In brief, hiPSC-CMs were cultured under hyperglycemic conditions (25 mM glucose) and treated for 24 h with vehicle, alpelisib (100 nM), fulvestrant (100 nM), or the combination of alpelisib and fulvestrant, in the absence or presence of dapagliflozin (1 μM), as described above. At the end of the treatment period, both cell lysates and culture supernatants were collected to quantify intracellular inflammasome components and secreted cytokines.

#### 4.6.1. Intracellular Inflammasome-Related Proteins

Intracellular levels of NLRP3 (Human NLRP3 ELISA Kit (OKEH03368), Aviva Systems Biology, San Diego, CA, USA) and MyD88 (Human MyD88 ELISA Kit (ab171341), Abcam, Milan, Italy) were measured in cardiomyocyte lysates using human-specific enzyme-linked immunosorbent assays (ELISAs) according to the manufacturers’ instructions. Briefly, cells were washed with cold phosphate-buffered saline (PBS) and lysed using ice-cold lysis buffer supplemented with protease inhibitors. Lysates were clarified by centrifugation (12,000× *g*, 10 min, 4 °C) to remove cellular debris. Supernatants were collected and used for ELISA quantification [70]. Samples and standards were incubated in antibody-coated microplates to allow antigen binding. After washing steps to remove unbound material, detection antibodies and substrate solution were sequentially added. Absorbance was measured at 450 nm using a microplate reader, and concentrations were calculated using a four-parameter logistic (4-PL) standard curve generated for each plate. Because intracellular protein abundance may vary depending on cell number and treatment-related cytotoxicity, NLRP3 and MyD88 levels were normalized to total protein content determined from parallel lysates using a bicinchoninic acid (BCA) protein assay [71]. Data are expressed as protein concentration relative to total cellular protein (e.g., pg/mg protein).

#### 4.6.2. Secreted Cytokines and Chemokines

To evaluate inflammatory mediator release from cardiomyocytes, the concentrations of IL-1β (Human IL-1 beta ELISA Kit (A270338) Antibodies, Bromma, Sweden), IL-18 (Human IL-18 ELISA Kit (A3483), Antibodies, Bromma, Sweden), IL-6 (ab178013,AbCam, Milan, Italy), TNF-α (ab181421, AbCam, Milan, Italy) and CCL2 (MCP-1, ab179886, AbCam, Milan, Italy) were quantified in culture supernatants using human-specific ELISA kits following the manufacturers’ protocols [72,73]. Conditioned media were collected at the experimental endpoint and clarified by centrifugation (300–500× *g*, 5 min, 4 °C) to remove residual cellular debris. Supernatants were aliquoted and stored at −80 °C until analysis to avoid repeated freeze–thaw cycles. Standards and samples were loaded into pre-coated 96-well plates and incubated to allow antigen binding. After washing steps, detection antibodies and substrate solution were added sequentially, and absorbance was measured at 450 nm using a microplate reader. Cytokine concentrations were determined from standard curves generated for each assay. Because extracellular cytokine levels in vitro may be influenced by differences in cell number and viability, cytokine concentrations were normalized to total cellular protein content per well determined from parallel lysates using the BCA assay (Sigma Aldrich, Milan, Italy). Results are therefore expressed as normalized cytokine release relative to total cellular protein to account for variations in cell density across experimental conditions. All measurements were performed in duplicate and derived from independent biological replicates.

### 4.7. Mitochondrial Membrane Potential (ΔΨm)

Mitochondrial membrane potential (ΔΨm) was assessed using the potentiometric dye tetramethylrhodamine methyl ester (TMRM) in non-quench mode (Cat. N ab228569; AbCam, Milan, Italy). TMRM accumulates in mitochondria in a membrane potential-dependent manner and therefore serves as a sensitive indicator of mitochondrial polarization. Human iPSC-derived cardiomyocytes were incubated with TMRM (25–50 nM) for 30 min at 37 °C in phenol red-free assay buffer according to established protocols [74]. After incubation, cells were gently washed to remove excess dye and fluorescence was immediately measured using a microplate reader in order to minimize dye redistribution artifacts. TMRM fluorescence intensity is proportional to mitochondrial membrane polarization; therefore, a reduction in fluorescence signal reflects mitochondrial depolarization. To confirm assay responsiveness, the mitochondrial uncoupler FCCP (Sigma Aldrich, Milan, Italy) was used in separate wells as a positive control for mitochondrial membrane depolarization. For graphical representation, mitochondrial membrane potential values were expressed as ΔΨm relative to those of vehicle-treated control cells. Specifically, fluorescence values obtained from treated cells were normalized to control conditions and expressed as the change in membrane potential relative to control baseline. In this representation, negative ΔΨm values indicate a decrease in mitochondrial membrane potential (mitochondrial depolarization) compared with untreated control cells, reflecting mitochondrial dysfunction induced by pharmacological treatments [75]. All measurements were performed in independent biological replicates and normalized to vehicle-treated controls within each experimental condition.

### 4.8. Caspase-3 Activity Assay

Caspase-3 activation was quantified to assess apoptotic signaling in human iPSC-derived cardiomyocytes following experimental treatments. Caspase-3 activation was selected as a marker of execution-phase apoptosis in cardiomyocytes, as this protease represents a central effector of the apoptotic cascade responsible for the cleavage of multiple structural and regulatory proteins during programmed cell death [76]. At the experimental endpoint, cell lysates were collected and clarified by centrifugation to remove cellular debris. Caspase-3 levels were determined using a Caspase 3 Cell-Based ELISA Kit (A102907, AbCam, Milan, Italy) according to the manufacturer’s instructions. Briefly, standards and samples were added to antibody-coated microplates and incubated to allow antigen binding. After washing steps to remove unbound proteins, detection antibodies and substrate solution were sequentially added. Absorbance was measured at 450 nm using a microplate reader, and caspase-3 concentrations were calculated using a four-parameter logistic (4-PL) standard curve generated for each plate. Because protein abundance in cell lysates may vary depending on cell number and treatment-related cytotoxicity, caspase-3 levels were normalized to total protein content determined in parallel lysates using a bicinchoninic acid (BCA) protein assay. Results are therefore expressed as picograms of caspase-3 per milligram of total protein (pg/mg protein) to account for potential differences in cell density and viability across experimental conditions. All samples were analyzed in duplicate and data were obtained from independent biological replicates.

### 4.9. Cardiac Injury Biomarkers

To assess cardiomyocyte-specific injury with translational relevance, cardiac troponin I (cTnI) and heart-type fatty acid binding protein (H-FABP/FABP3) were quantified in culture supernatants [77]. Cardiac troponin I is a well-established biomarker of myocardial injury widely used in clinical practice, and its extracellular release in cell culture systems reflects sarcolemmal damage and cardiomyocyte injury. H-FABP is a small cytosolic protein rapidly released following cardiomyocyte injury and is considered an early biomarker of myocardial damage. Conditioned media were collected at the experimental endpoint (24 h), clarified by centrifugation (300–500× *g*, 5 min, 4 °C) to remove cellular debris, aliquoted, and stored at −80 °C until analysis (single freeze–thaw cycle). Troponin I concentrations were measured using Human Cardiac Troponin I ELISA Kit (ab200016, AbCam, Milan, Italy) according to the manufacturer’s instructions [78]. To complement troponin measurements, H-FABP levels were determined in the same supernatants using Human H-FABP ELISA Kit (ab243682, AbCam, Milan, Italy) validated for cell culture samples. Standards and samples were analyzed in duplicate and absorbance was measured at 450 nm using a microplate reader. Concentrations were calculated using a four-parameter logistic (4-PL) standard curve generated for each plate. Because extracellular biomarker levels in vitro may be influenced by differences in cell number and viability, troponin I concentrations measured in culture supernatants were normalized to total protein content per well determined from parallel cell lysates using a bicinchoninic acid (BCA) assay. Accordingly, troponin release is expressed as picograms of troponin I per milligram of total protein (pg/mg protein). H-FABP concentrations were similarly normalized to total protein content to account for potential variations in cell density across experimental conditions.

### 4.10. Late Sodium Current (I_NaL) Recordings in hiPSC-Derived Cardiomyocytes

Late sodium current (I_NaL) was measured in human induced pluripotent stem cell-derived cardiomyocytes (hiPSC-CMs; ventricular-like phenotype) using the whole-cell patch-clamp technique in voltage-clamp configuration at room temperature (22–23 °C). Cells were plated on Matrigel-coated glass coverslips and used after 5–7 days of post-thaw stabilization, when stable morphology and spontaneous contractile activity were observed. To reproduce the metabolic conditions associated with PI3Kα inhibitor-induced dysglycemia, hiPSC-CMs were cultured under hyperglycemic conditions (25 mM D-glucose) for 72 h prior to drug exposure and maintained in the same glucose concentration throughout the experiment. Cells were then treated for 24 h with vehicle (0.1% DMSO), alpelisib (100 nM), fulvestrant (100 nM), or their combination, in the absence or presence of dapagliflozin (1 μM). Dapagliflozin was added 60 min before alpelisib/fulvestrant and maintained throughout the incubation period. Whole-cell currents were recorded using a low-noise patch-clamp amplifier. Patch pipettes (2–4 MΩ) were filled with an intracellular solution containing (in mM): CsF 110, CsCl 20, NaF 5, EGTA 10, and HEPES 10 (pH 7.4 adjusted with CsOH). The external bath solution contained (in mM): NaCl 135, MgCl_2_ 1.0, glucose 10, and HEPES 10 (pH 7.4 adjusted with CsOH). To minimize contamination from calcium and potassium currents, nisoldipine (1 μM), NiCl_2_ (200 μM), and 4-aminopyridine (500 μM) were added to the bath solution. Currents were digitized at 20–50 kHz and filtered at 5–10 kHz. Series resistance was compensated by ≥70%, and cells were excluded if series resistance varied by more than 20% during recording. I_NaL was elicited using a 200-ms depolarizing pulse from a holding potential of −120 mV to −30 mV applied every 2–5 s. Peak sodium current was defined as the maximal inward current during the initial transient phase. Late sodium current was quantified as the mean current measured in a 3-ms window at 195–198 ms after the onset of the depolarizing pulse and expressed as the percentage of peak sodium current (|I_NaL|/|I_Na,peak| × 100). Each experimental group included cells obtained from at least three independent culture preparations [79]. Data were analyzed offline using the electrophysiological analysis software Clampfit 10.7 (Molecular Devices, San Jose, CA, USA).

### 4.11. Statistical Analysis

Data were obtained from six independent biological replicates per condition (independent wells) and experiments were repeated independently. Results are presented as mean ± SEM. Statistical comparisons among experimental groups were performed using two-way analysis of variance (ANOVA) to account for the effects of treatment conditions and pharmacological interventions, followed by Tukey’s multiple-comparison post hoc test to correct for multiple testing. Pre-specified pairwise comparisons were conducted to evaluate (i) drug-induced cardiomyocyte injury relative to untreated controls and (ii) the cardioprotective effect of dapagliflozin relative to the corresponding drug-treated groups. A *p* value < 0.05 was considered statistically significant. All statistical analyses and graph generation were performed using GraphPad Prism software version 8.4.3 (686) (GraphPad Software, San Diego, CA, USA).

## 5. Conclusions

This study provides evidence that combined PI3Kα inhibition and estrogen receptor blockade under hyperglycemic conditions are associated with a cardiotoxic phenotype in human cardiomyocytes characterized by mitochondrial dysfunction, oxidative membrane damage, inflammasome-associated signaling, inflammatory cytokine signaling, and increased release of cardiac injury biomarkers. These findings suggest a previously underrecognized cell-autonomous mechanism of cardiometabolic toxicity associated with PI3Kα-targeted therapy in precision oncology. Importantly, the SGLT2 inhibitor dapagliflozin markedly attenuated these pathological responses, preserving mitochondrial integrity, reducing lipid peroxidation, suppressing inflammasome-related inflammatory signaling, and limiting cardiomyocyte injury. These results support a potential cardioprotective role of dapagliflozin that may extend beyond systemic glucose lowering and involve modulation of oxidative and inflammatory stress pathways within cardiomyocytes. Collectively, our findings highlight oxidative stress, mitochondrial dysfunction, and inflammasome-associated signaling as key interconnected mechanistic nodes linking PI3Kα inhibitor-induced metabolic toxicity to myocardial injury. These data provide a mechanistic rationale for investigating SGLT2 inhibitors as cardiometabolic protective strategies in patients receiving PI3Kα-targeted therapies for breast cancer. Future translational and clinical studies will be required to determine whether SGLT2 inhibition can simultaneously improve metabolic control, enhance cardiovascular safety, and support therapeutic durability in patients treated with PI3K pathway inhibitors.

## Data Availability

The datasets analyzed for this study can be found in the Zenodo repository at https://zenodo.org/records/18979057 (accessed on 12 March 2026).

## Abbreviations

The following abbreviations are used in this manuscript:

**4**-HNE	4-Hydroxynonenal.
**A**kt	Protein kinase B.
**A**NOVA	Analysis of Variance.
**B**CA	Bicinchoninic Acid.
**C**CL2	C-C Motif Chemokine Ligand 2.
**c**TnI	Cardiac Troponin I.
**Δ**Ψm	Mitochondrial Membrane Potential.
**D**MSO	Dimethyl Sulfoxide.
**E**LISA	Enzyme-Linked Immunosorbent Assay.
**E**R	Estrogen Receptor.
**F**AERS	FDA Adverse Event Reporting System.
**F**CCP	Carbonyl Cyanide-p-Trifluoromethoxyphenylhydrazone.
**G**LUT4	Glucose Transporter Type 4.
**H**-FABP	Heart-Type Fatty Acid Binding Protein.
**H**EPES	4-(2-Hydroxyethyl)-1-Piperazineethanesulfonic Acid.
**H**ER2	Human Epidermal Growth Factor Receptor 2.
**h**iPSC-CMs	Human Induced Pluripotent Stem Cell-Derived Cardiomyocytes.
**H**R+	Hormone Receptor-Positive.
**I**L	Interleukin.
**i**PSC	Induced Pluripotent Stem Cell.
**I**_na_L/I_NaL	Late Sodium Current.
**M**CP-1	Monocyte Chemoattractant Protein-1.
**M**DA	Malondialdehyde.
**m**TORC1	Mechanistic Target of Rapamycin Complex 1.
**MTS**	**3-(4:5**-Dimethylthiazol-2-yl)-5-(3-Carboxymethoxyphenyl)-2-(4-Sulfophenyl)-2H-Tetrazolium.
**M**yD88	Myeloid Differentiation Primary Response 88.
**N**F-κB	Nuclear Factor Kappa B.
**N**LRP3	NLR Family Pyrin Domain Containing 3.
**P**BS	Phosphate-Buffered Saline.
**P**I3K	Phosphatidylinositol 3-Kinase.
**P**IK3CA	Phosphatidylinositol-4,5-Bisphosphate 3-Kinase Catalytic Subunit Alpha.
**P**L 4-PL	Four-Parameter Logistic.
**R**OS	Reactive Oxygen Species.
**S**EM	Standard Error of the Mean.
**S**ERCA2	Sarco/Endoplasmic Reticulum Ca2+-ATPase 2.
**S**GLT2	Sodium–Glucose Cotransporter 2.
**T**BARS	Thiobarbituric Acid Reactive Substances.
**T**BA	Thiobarbituric Acid.
**T**MRM	Tetramethylrhodamine Methyl Ester.
**T**NF-α	Tumor Necrosis Factor Alpha.
**U**TIC	Unità di Terapia Intensiva Cardiologica.
